# Association of Non-Steroidal Anti-Inflammatory Drugs, Genetic Risk, and Environmental Risk Factors with Incidence of Colorectal Cancer

**DOI:** 10.3390/cancers14205138

**Published:** 2022-10-20

**Authors:** Jiaojiao Ren, Peidong Zhang, Zhihao Li, Xiru Zhang, Wenfang Zhong, Weiqi Song, Xing Wang, Pingming Gao, Chen Mao

**Affiliations:** 1 Affiliated Foshan Maternity & Child Healthcare Hospital, Southern Medical University, Foshan 528000, China; 2 Department of Epidemiology, School of Public Health, Southern Medical University, Guangzhou 510515, China; 3 Department of Neurosurgery, Nanfang Hospital, Southern Medical University, Guangzhou 510515, China; 4 The Second School of Clinical Medicine, Southern Medical University, Guangzhou 510280, China; 5 Microbiome Medicine Center, Department of Laboratory Medicine, Zhujiang Hospital, Southern Medical University, Guangzhou 510280, China

**Keywords:** colorectal cancer, non-steroidal anti-inflammatory drugs, polygenic risk score, environmental risk factors

## Abstract

**Simple Summary:**

It remains unknown whether regular use of non-steroidal anti-inflammatory drugs (NSAIDs) could attenuate the impact of genetic risk and environmental risk factors on colorectal cancer (CRC). In the current study, we evaluated the association of NSAID use, genetic risk, and environmental risk factors with CRC incidence using data from a UK Biobank cohort. We found that regular use of NSAIDs was associated with a lower risk of CRC. Although there were no statistical interactions observed between NSAID use, environmental risk and genetic risk, regular NSAID use was still associated with lower CRC incidence among subjects with either high environmental risk or high genetic risk. Furthermore, low environmental risk was associated with lower CRC incidence among subjects with high genetic risk. These findings emphasized the importance of the chemopreventive effect of regular use of NSAIDs and controlling modifiable environmental risk factors to reduce CRC incidence, even among individuals with a moderate or high genetic risk of CRC.

**Abstract:**

Regular use of non-steroidal anti-inflammatory drugs (NSAIDs) was associated with the lower risk of colorectal cancer (CRC). However, whether regular use of NSAIDs could attenuate the effect of genetic risk and environmental risk factors on CRC is unknown. We aimed to evaluate the association of NSAID use, genetic risk, and environmental risk factors with CRC. Using data from a UK Biobank, a Cox proportional hazards model was performed to estimate the risk of CRC according to NSAID use, polygenic risk score, and environmental risk factors. Regular use of NSAIDs was associated with a 36.0% lower risk of CRC. No statistically significant interaction was observed between NSAID use and the genetic risk score (*p* = 0.190), and between NSAID use and the environmental risk score (*p* = 0.740). However, regular NSAID use was still associated with lower CRC incidence among subjects with either high environmental risk or high genetic risk. Furthermore, the genetic and environmental risk of CRC were additives. These findings appear to support the chemopreventive effect of regular NSAID use. Furthermore, controlling of modifiable environmental risk factors can reduce the CRC risk, especially among individuals with a moderate or high genetic risk of CRC.

## 1. Introduction

Colorectal cancer (CRC) is the third most common type of cancer and the second most common cause of cancer-related deaths worldwide. CRC accounted for approximately 1.9 million new cases and 940,000 deaths in 2020 [1]. Consequently, it would be more important to focus on the risk reduction in CRC incidence and mortality through prevention interventions, including screening, early detection and chemoprevention strategies [2]. The colonoscopy screening has shown a greatly reduced association of CRC incidence and mortality through the removal of precancerous polyps, but low adherence and the rising costs of health care are challenges [3,4]. The chemoprevention may be a good strategy aimed at reducing CRC incidence and mortality [5].

Numerous studies have suggested that regular use of non-steroidal anti-inflammatory drugs (NSAIDs) was associated with the lower risk of CRC [6,7,8]. However, gastrointestinal complications and cardiovascular events related to NSAID use made it important to identify the individuals whose benefit outweighs the risk [9]. Individual differences in the effect of NSAID use on CRC are partly due to germline variation [10,11]. The most recent genome-wide association studies (GWASs) have identified large numbers of single-nucleotide polymorphisms (SNPs), which were associated with the risk of CRC [12]. However, the effects of each common genetic variant were small or moderate. The polygenic risk score (PRS) is a continuous method of assessing the cumulative effect of genetic variants, which predicts a higher risk of CRC [13].

Additionally, several risk factors, including obesity, low-quality diet, smoking tobacco, excessive alcohol consumption, irregular physical activity, occupational exposure (asbestos, or benzene), and history of Type 2 diabetes have been found to be associated with CRC risk [14,15,16]. A few studies have reported on the association between combined lifestyle factors and genetic risk with CRC [17,18], however, it is unclear whether regular use of NSAIDs could attenuate the influence of genetic risk and environmental risk factors on CRC.

The purpose of this study was to evaluate the potential effect of regular NSAID use on the associations of the PRS and environmental risk factors with CRC incidence using a population-based cohort study of the UK Biobank.

## 2. Materials and Methods

### 2.1. Study Participants

The UK Biobank recruited over 500,000 individuals who attended 1 of 22 assessment centers throughout the UK from 2006 to 2010. Health-related information of participants was obtained through self-administered questionnaires, interviews, and physical assessments. Individuals were asked whether they regularly took NSAIDs, including aspirin or ibuprofen. Regular use of NSAIDs was defined as taking them in most days of the week for the last 4 weeks. Participants were excluded from this study if they withdrew from the study, their genotype information did not meet quality control requirements, they had a second degree or closer relative, they had cancer history or inflammatory bowel diseases, they were of non-White/European descent, they had the missing environmental risk data, or they had the BMI < 18.5 kg/m^2^ (see Supplementary Figure S1). The study was approved by the research ethics committee (UK Biobank 11/NW/0382) and all individuals provided informed consent prior to involvement.

### 2.2. Polygenic Risk Score

More details on the genotyping process and arrays used in the UK Biobank study have been previously described [19]. In this study, the PRS was constructed based on genetic variants identified to be associated with the risk of CRC in GWASs in participants of European ancestry [20]. We excluded participants with genotype data failing to meet quality control (low call rates, or outliers for heterozygosity) and selected individuals who were of White/European descent for analyses. After imputation, a total of 139 independently related SNPs could be extracted from the UK Biobank (see Supplementary Table S1). We built the PRS by summing the number of risk alleles weighted by β coefficients for each SNP using PLINK version 1.9 (population linkage, Menlo Park, CA, USA) [21]. Derivation of the PRS has been previously described in detail [22]. This score was z-standardized and quintiles were divided into three groups: low (quintile 1), intermediate (quintiles from 2 to 4), and high (quintile 5) risk.

### 2.3. Environmental Risk Score

Environmental risk factors included demographic, lifestyle, diet, as well as physical traits and medical history. These data were collected using a touchscreen questionnaire at baseline. According to the previous study [23], the environmental risk score was calculated based on 8 risk factors of CRC, including education, diet (intake of fruits, vegetables, whole grains, low-fat dairy products, red or processed meats, sugar-sweetened beverages, and sodium), physical activity, smoking status and smoking pack years, alcohol consumption, occupational exposure, history of type 2 diabetes, and body mass index (BMI). The definitions of environmental risk factors in the UK Biobank are provided in Supplementary Table S2. Participants scored 1 point for the low-risk environmental factor of CRC based on recommendations (see Supplementary Table S3). The environmental risk scores ranged from 0 to 8, with higher scores indicating a lower risk of CRC. A weighted environmental risk score was derived from the β coefficients of each environmental risk factor according to the Cox proportional hazards model adjusted for sex, age, household income, Townsend deprivation index, family history of CRC, screening history of CRC, NSAID use, relatedness of individuals, genotyping chip, and the first 20 principal components of ancestry. We multiplied the unweighted environmental risk scores by β coefficients, divided the sum of the β coefficients and then multiplied it by 100. The weighted environmental risk score was defined as low, intermediate, and high risk based on the similar distribution of the unweighted environmental risk score [24].

### 2.4. Ascertainment of CRC Incidence

Information on the diagnosis of CRC was obtained from the National Health Service (NHS) Information Center and the NHS Central Register of Scotland (follow-up time through 25 February 2018, for England and Wales, and through 31 October 2016, for Scotland). Individuals with CRC were identified based on their first diagnosed cancer according to the International Classification of Diseases, 9th or 10th Revisions (see Supplementary Table S4).

### 2.5. Statistical Analyses

The distribution of the characteristics of the study population according to CRC status was evaluated by descriptive analysis method. Multiple imputations were performed to impute missing covariate values (12.6% for household income and 0.11% for Townsend deprivation index). A Cox proportional hazards model was conducted to assess the relationship of CRC between NSAID use, genetic risk, and environmental risk, which was adjusted for sex, age, household income, Townsend deprivation index, family history of CRC, screening history of CRC, relatedness of individuals, genotyping chip, and the first 20 principal components of ancestry. The assumption of proportional hazards was tested by Schoenfeld residuals [25], and the inverse assumption was not observed. Moreover, the multiplicative interaction by adding a cross-product term through the adjusted model between NSAID use and the PRS, and NSAID use and the weighted environmental risk score, respectively.

In addition, sensitivity analyses were evaluated after excluding participants who had third-degree relatedness, developed outcomes within the first 2 years, and with missing covariate data. Subgroup analyses were conducted by sex (male or female), age (<60 years or ≥60 years), and screening history of CRC (yes or no).

A population-attributable fraction (PAF) was calculated, which was an estimate of the proportion of CRC incidence that could be prevented if all participants would regularly use NSAIDs [26]. Two-sided *p* values < 0.05 were considered statistically significant. All statistical analyses were performed using R v4.1.0 (R Foundation for Statistical Computing, Vienna, Austria).

## 3. Results

A total of 336 017 White/European participants were included in this study (mean [standard deviation] age, 56.5 [8.0] years; 52.7% were female). Over 2,947,705 person years of follow-up (median [interquartile range], 8.8 [8.2–9.6] years), 2942 cases of CRC were documented. The characteristics of eligible participants are presented in Table 1. Compared to non-CRC cases, individuals who developed CRC were more likely to be older, male, with a lower household income, with a family history of CRC, undergo fewer CRC screenings, non-regular use of NSAIDs and have higher genetic and environmental risk.

### 3.1. Relationships of NSAID Use, Genetic Risk, Environmental Risk Factors and Population-Attributable Fractions with CRC Incidence

The regular use of NSAIDs was associated with a lower risk of CRC incidence (HR: 0.64, 95% CI: 0.59–0.70). Compared with participants who were with a low genetic risk, participants with a high genetic risk had a greater risk of CRC incidence (HR: 3.28, 95% CI: 2.88–3.74; P_trend_ < 0.001). Among those who were with a low environmental risk, participants with a high environmental risk were associated with the increased risk of CRC incidence (HR: 1.38, 95% CI: 1.25–1.52; P_trend_ < 0.001). Regarding the population-attributable fractions, if all individuals would regularly use NSAIDs, 21.1% (95% CI: 16.2–25.8%) of incident CRC cases could be prevented (Table 2).

### 3.2. Relationship of Environmental Risk Factors with CRC Incidence According to the Genetic Risk

When we examined the association of each environmental risk factor with CRC incidence risk, lower qualification, a higher BMI, current smoking, excessive alcohol consumption, unhealthy diet, irregular physical activity, occupational exposure and history of type 2 diabetes were associated with a higher risk of CRC incidence (see Supplementary Table S5).

For the weighted environmental risk score, 37.9% of participants were classified as having a low environmental risk, 42.3% intermediate environmental risk, and 19.8% high environmental risk. In the intermediate and high genetic risk category, participants with a high environmental risk were associated with an increased risk of CRC incidence compared to a low environmental risk, and the HRs were 1.34 (95% CI, 1.18–1.53) and 1.54 (95% CI, 1.30–1.83), respectively (see Supplementary Table S6). When combining genetic risk and environmental risk factors, the HR of participants with a high genetic and high environmental risk was 4.18 (95% CI, 3.32–5.26) times that of those with a low genetic and low environmental risk (see Supplementary Table S7). 

### 3.3. Relationship of Genetic Risk and Environmental Risk Factors with CRC Incidence Stratified by Non-Regular and Regular NSAID Use

Table 3 presents the relationship of genetic risk categories with CRC incidence according to NSAID use. Overall, non-regular NSAID users had a higher risk of CRC incidence than regular NSAID users. Furthermore, there was a continuously increasing trend of CRC incidence risk according to the increase in the genetic risk, regardless of the NSAID use. No interaction effect was observed between NSAID use and the PRS (*p* = 0.190). 

Table 4 shows the relationship of environmental risk categories with CRC incidence according to NSAID use. In general, the CRC incidence risk were higher in participants with non-regular use of NSAIDs than in those who had regular use of NSAIDs. Moreover, the CRC incidence risk consistently increased depending on the increase in the environmental risk, regardless of NSAID use. We found no interaction effect between NSAID use and the weighted environmental risk score (*p* = 0.740).

**Figure 1:**
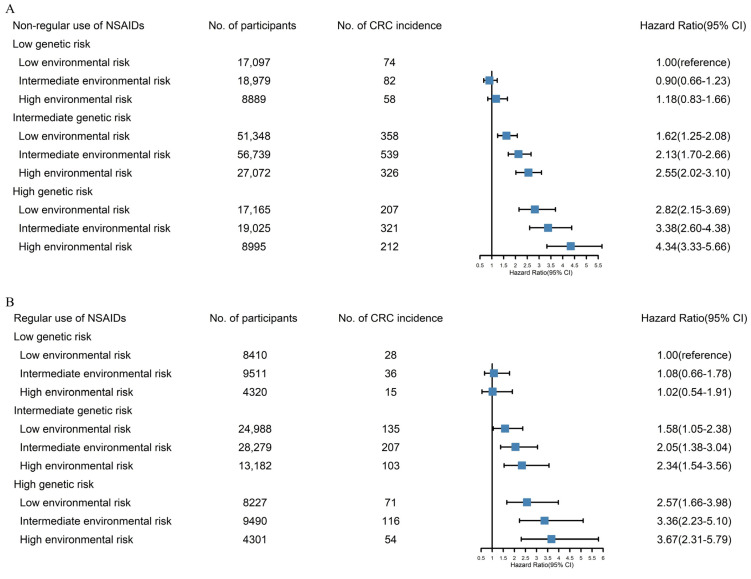
The CRC incidence risk in accordance with genetic risk and environmental risk according to NSAID use: (**A**) non-regular use of NSAIDs; (**B**) regular use of NSAIDs.

When genetic risk and environmental risk factors were combined and analyzed by non-regular and regular NSAID use, the HR of participants with a high genetic risk, high environmental risk, and non-regular use of NSAIDs was 4.34 (95% CI: 3.33–5.66) compared with participants with a low genetic risk, low environmental risk, and non-regular use of NSAIDs. Moreover, the HR of participants with a high genetic risk, low environmental risk, and non-regular use of NSAIDs was 2.82 (95% CI: 2.15–3.69) compared with participants with a low genetic risk, low environmental risk, and non-regular use of NSAIDs. By comparison, the HR of participants with a high genetic risk, low environmental risk, and regular use of NSAIDs was 2.57 (95% CI: 1.66–3.98) compared with participants with a low genetic risk, low environmental risk, and regular use of NSAIDs (Figure 1).

For a series of sensitivity analyses, the same pattern of associations was observed after excluding related individuals, events occurred within the first 2 years and individuals with missing covariate data (see Supplementary Table S8). We conducted subgroup analyses by the following factors: sex, age, and screening history of CRC. For non-regular NSAID users in a high genetic and high environmental risk, participants were men, younger (age < 60 years) and with no screening history had an increased risk of CRC than those who were non-regular NSAID users in a low genetic and low environmental risk (see Supplementary Tables S9 and S10). Similar to our main results, with the elevated environmental risk, the risk of CRC incidence increased gradually, regardless of the NSAID use.

## 4. Discussion

Based on our cohort study of the UK Biobank, we investigated the association of NSAID use, genetic risk, and environmental risk factors with CRC incidence. Genetic risk and environmental risk factors played a comparably important role in CRC incidence in both regular and non-regular NSAID users. In any genetic risk category, individuals with a higher environmental risk had a higher risk of CRC incidence than those who had a low environmental risk. That revealed that the effects of the increased genetic risk on CRC incidence could be attenuated to a certain extent. When the genetic risk and environmental risk factors were combined, individuals with a high genetic risk and environmental risk had an additive risk of CRC incidence. 

Previous studies have elucidated the effects of genetic or environmental risk on CRC incidence [27,28]. Recently, Chen et al.’s study evaluated the association of NSAID use with CRC incidence at different levels of the PRS based on 140 risk variants [29]. However, environmental risk factors associated with the risk of CRC were not included in that study, which could not assess the influence of NSAID use on the association of environmental risk factors and the PRS with CRC incidence. To the best of our knowledge, our study is the first to comprehensively investigated the association of CRC incidence on the basis of NSAID use, 139 genetic risk variants, and 8 environmental risk factors. We showed that environmental risk factors could modify the association of genetic risk with CRC incidence. Individuals might be conscious of the irreversibly increased genetic risk with CRC incidence, but our study showed that a lower environmental risk was still associated with the lower risk of CRC incidence.

In this study, regular use of NSAIDs was associated with 36% lower risk of CRC incidence, which was in line with the findings of the previous studies [30,31]. NSAIDs that inhibit the activity of cyclooxygenase-2 and form prostaglandin E_2_ subsequently have been recognized as the main chemopreventive mechanism [32]. In addition, NSAIDs may play a significant role in CRC incidence through NF-kappaB or PI3K signaling pathway [33,34]. However, the risk of adverse drug reactions attributed to the long-term use of NSAIDs has aroused great concern [35]. Therefore, identifying subgroups who may benefit from regular use of NSAIDs can contribute to optimize the benefit risk ratio of CRC chemoprevention. A randomized controlled trial (RCT) reported that healthy participants aged 70 years or older who daily took low doses of aspirin (100 mg) were not associated with the risk of CRC incidence, but an increased risk of CRC mortality [36]. These potential differences might be explained by the variability of the age in the population [37]. Our study showed that among regular NSAID use group, the younger population (age < 60 years) in a high genetic and high environmental risk had a higher risk of CRC incidence than the older population with the same genetic and environmental risk (age ≥ 60 years). A prospective cohort study suggested that the benefit was most apparent after approximately 10 years since initiation of aspirin [38]. Another RCT found that the best start time of regular NSAID use was under 50 years (about 10 years), regarding the delay of the effect on NSAIDs chemoprevention [39].

We found that individuals with a high genetic risk, high environmental risk, with regular use of NSAIDs and screening history had the lowest risk of CRC incidence. This implied that screening history had an essential protective effect against CRC incidence, which was consistent with established evidence [40]. The precursor lesions and adenomas can be detected and removed by colonoscopy, which reduce the risk of CRC incidence [41]. Further studies should elucidate the association of regular NSAID use and screening history with CRC incidence.

The strengths of our study included a prospective cohort design; large sample size; and the availability of information on demographic, genetic risk, environmental risk factors, and NSAID use. Furthermore, we incorporated 139 genetic risk variants previously reported and added 8 environmental risk factors to investigate the association of NSAID use, genetic risk, and environmental risk factors with CRC incidence. However, this study has some limitations. Firstly, no information was available on the dose, frequency or duration of regular NSAID use, and we could not make a more comprehensive evaluation of regular NSAID use on CRC incidence. Secondly, some environmental risk factors and the NSAID use were self-reported and measured once, thus misclassification errors might have biased these findings. Thirdly, the present analyses did not consider the changes in environmental risk factors over time, which might lead to deviations in the precise exposure to environmental risk factors. Fourthly, the weight of weighted environmental risk scores were derived from the regression coefficients of the Cox proportional hazards model in this cohort, which might result in a risk of overfitting the effect. Fifthly, the numbers of cases was relatively small in subgroups and did not have sufficient power to clarify the association. Finally, study samples were selected from participants of European descent, and this might reduce the generalizability of these findings to other races.

## 5. Conclusions

In conclusion, regular use of NSAIDs has a strong protective effect on CRC incidence. The risk attributable to the genetic and environmental risk of CRC were additive, which was independent of NSAID use. These findings appear to support the chemopreventive effect of regular NSAID use. Furthermore, controlling of modifiable environmental risk factors can reduce the CRC risk, especially among individuals with a moderate or high genetic risk of CRC. Further studies precisely identifying the subgroup populations with the highest benefit–risk ratio for regular NSAID use are warranted.

## Figures and Tables

**Table 1:** Comparison of characteristics of participants with and without CRC ^a^.

Characteristics	Incident CRC (*n* = 2942) No. (%)	Non-Incident CRC (*n* = 333,075) No. (%)	*p* Value
Age, mean (SD), years	60.8 (6.4)	56.5 (8.0)	<0.001
Male, *n* (%)	1752 (59.6)	157,299 (47.2)	<0.001
Education, *n* (%)			0.004
Lower qualification	1522 (51.7)	163,397 (49.1)	
Higher qualification	1420 (48.3)	169,678 (50.9)	
Household income, *n* (%), GBP			<0.001
<18,000	697 (23.7)	64,805 (19.5)	
18,000–30,999	868 (29.5)	86,547 (26.0)	
31,000–51,999	785 (26.7)	94,591 (28.3)	
52,000–100,000	466 (15.8)	69,973 (21.0)	
>100,000	126 (4.3)	17,159 (5.2)	
Townsend deprivation index, median (IQR)	−2.4 (−3.8, 0.1)	−2.3 (−3.7, 0.2)	0.056
Screening history of CRC, *n* (%)	730 (24.8)	88,931 (26.7)	0.021
Family history of CRC, *n* (%)	419 (14.2)	35,659 (10.7)	<0.001
BMI, *n* (%), kg/m^2^			<0.001
18.5–24.9	833 (28.3)	109,131 (32.7)	
25.0–29.9	1333 (45.3)	143,092 (43.0)	
≥30.0	776 (26.4)	80,852 (24.3)	
DASH score, mean (SD)	23.3 (3.1)	23.5 (3.1)	0.008
Smoking, *n* (%)			<0.001
Never or former (<30 pack years)	2114 (71.9)	270,906 (81.3)	
Current or former (≥30 pack years)	828 (28.1)	62,169 (18.7)	
Alcohol consumption, *n* (%), g/day			<0.001
0	423 (14.4)	50,005 (15.0)	
Men: 0.1–28; women: 0.1–14	1894 (64.4)	224,262 (67.3)	
Men: >28; women: >14	625 (21.2)	58,808 (17.7)	
Occupational exposure, *n* (%)			<0.001
Rarely/never	2335 (79.3)	274,679 (82.5)	
Sometimes	529 (18.0)	51,330 (15.4)	
Often	78 (2.7)	7066 (2.1)	
Regular physical activity, *n* (%)	1492 (50.7)	178,243 (53.5)	0.002
History of type 2 diabetes, *n* (%)	220 (7.5)	16,660 (5.0)	<0.001
Regular use of NSAIDs, *n* (%)	765 (26.0)	109,943 (33.0)	<0.001
Genetic risk category, *n* (%)			<0.001
Low	293 (10.0)	66,913 (20.1)	
Intermediate	1668 (56.7)	199,940 (60.0)	
High	981 (33.3)	66,222 (19.9)	
Environmental risk category, *n* (%)			<0.001
Low	873 (29.7)	126,362 (37.9)	
Intermediate	1301 (44.2)	140,722 (42.3)	
High	768 (26.1)	65,991 (19.8)	

^a^ CRC, colorectal cancer; SD, standard deviation; IQR, interquartile range; BMI, body mass index; DASH score, dietary approaches to stop hypertension; NSAIDs, non-steroidal anti-inflammatory drugs.

**Table 2:** The multivariate-adjusted hazard ratio (95% CI) of risk factors associated with CRC ^a^.

	No. of Participants	No. of Cases (%) /Person Years	HR (95% CI) ^b^	*p* Value	*p* Value for Trend ^c^
Genetic risk categories					
Low	67,206	293 (0.44)/593,593	1 [reference]		<0.001
Intermediate	201,608	1668 (0.83)/1,781,989	1.87 (1.65–2.12)	<0.001	
High	67,203	981 (1.46)/572,123	3.28 (2.88–3.74)	<0.001	
Environmental risk categories					
Low	127,235	873 (0.69)/1,125,921	1 [reference]		<0.001
Intermediate	142,023	1301 (0.92)/1,253,727	1.20 (1.10–1.31)	<0.001	
High	66,759	768 (1.15)/568,057	1.38 (1.25–1.52)	<0.001	
Regular use of NSAIDs					
No	225,309	2177 (0.97)/1,965,614	1 [reference]		*--*
Yes	110,708	765 (0.69)/982,091	0.64 (0.59–0.70)	<0.001	
Population-attributable fraction (%)					
Regular use of NSAIDs	110,708	765 (0.69)/982,091	1 [reference]		*--*
Non-regular use of NSAIDs	225,309	2177 (0.97)/1,965,614	21.1 (16.2–25.8)	*--*	

^a^ CRC, colorectal cancer; CI, confidence interval; NSAIDs, nonsteroidal anti-inflammatory drugs. ^b^ Adjusted for age, sex, household income, Townsend deprivation index, family history of CRC, screening status, relatedness, genotyping chip, first 20 principal components of ancestry, genetic risk categories, environmental risk categories, and NSAID use. ^c^ Calculated considering the polygenic risk score and the weighted environmental risk score as continuous variables.

**Table 3:** The CRC incidence risk in accordance with genetic risk categories according to NSAID use ^a^.

NSAIDs Use	Genetic Risk	No. of Participants	No. of CRC Incidence (%)/Person Years	HR (95% CI) ^b^	*p* Value	*p* Value for Trend ^c^	*p* Value for Interaction
Non-regular use	Low risk	44,965	214 (0.48)/394,384	1 [reference]		<0.001	0.190
Non-regular use	Intermediate risk	135,159	1223 (0.90)/1,187,550	1.86 (1.61–2.15)	<0.001		
Non-regular use	High risk	45,185	740 (1.64)/383,680	3.33 (2.86–3.88)	<0.001		
Regular use	Low risk	22,241	79 (0.36)/199,209	1 [reference]		<0.001	
Regular use	Intermediate risk	66,449	445 (0.67)/594,439	1.85 (1.46–2.35)	<0.001		
Regular use	High risk	22,018	241 (1.09)/188,443	3.03 (2.35–3.90)	<0.001		

^a^ CRC, colorectal cancer; HR, hazard ratio; CI, confidence interval; NSAIDs, non-steroidal anti-inflammatory drugs. ^b^ Adjusted for age, sex, household income, Townsend deprivation index, family history of CRC, screening history of CRC, environmental risk categories, relatedness, genotyping chip, and first 20 principal components of ancestry. ^c^ Calculated considering the polygenic risk scores as continuous variables.

**Table 4:** The CRC incidence risk in accordance with environmental risk categories according to NSAID use ^a^.

NSAIDs Use	Environmental Risk	No. of Participants	No. of CRC Incidence (%)/Person Years	HR (95% CI) ^b^	*p* Value	*p* Value for Trend ^c^	*p* Value for Interaction
Non-regular use	Low risk	85,610	639 (0.75)/753,254	1 [reference]		<0.001	0.740
Non-regular use	Intermediate risk	94,743	942 (0.99)/829,898	1.25 (1.11–1.42)	0.001		
Non-regular use	High risk	44,956	596 (1.33)/382,462	1.46 (1.23–1.74)	<0.001		
Regular use	Low risk	41,625	234 (0.56)/372,667	1 [reference]		0.249	
Regular use	Intermediate risk	47,280	359 (0.76)/423,829	1.25 (0.91–1.71)	0.169		
Regular use	High risk	21,803	172 (0.79)/185,595	1.29 (1.03–1.62)	0.027		

^a^ CRC, colorectal cancer; HR, hazard ratio; CI, confidence interval; NSAIDs, non-steroidal anti-inflammatory drugs. ^b^ Adjusted for age, sex, household income, Townsend deprivation index, family history of CRC, screening history of CRC, genetic risk categories, relatedness, genotyping chip, and first 20 principal components of ancestry. ^c^ Calculated considering the weighted environmental risk scores as continuous variables.

## Data Availability

Data are available in a public, open access repository. The UK Biobank data are available from the UK Biobank on request (www.ukbiobank.ac.uk/ (accessed on 11 March 2022)).

## Supplementary Materials

The following supporting information can be downloaded at: https://www.mdpi.com/article/10.3390/cancers14205138/s1, Figure S1: Flow diagram of the study population selection; Table S1: List of single-nucleotide polymorphisms constructing the polygenic risk score for CRC; Table S2: Definitions of environmental risk factors in the UK Biobank; Table S3: Descriptions of environmental risk factors used to derive unweighted environmental risk score; Table S4: Definitions of diseases in the UK Biobank; Table S5: The hazard ratios of CRC incidence risk associated with each environmental risk factor and the environmental risk score; Table S6: The CRC incidence risk in accordance with environmental risk category within each genetic risk category; Table S7: The CRC incidence risk in accordance with the combined genetic risk and environmental risk; Table S8: The CRC incidence risk in accordance with the combined genetic and environmental risk according to NSAIDs use after excluding related participants, events occurred within the first 2 years and individuals with missing covariate data; Table S9: The CRC incidence risk in accordance with the combined genetic and environmental risk according to NSAIDs use by sex and age; Table S10: The CRC incidence risk in accordance with the combined genetic and environmental risk according to NSAIDs use by screening history of CRC. (References [42,43,44,45,46,47,48] are cited in the Supplementary Materials).

## Abbreviations

BMI	Body Mass Index
CRC	Colorectal Cancer
DASH	Dietary Approaches to Stop Hypertension
GWAS	Genome-Wide Association Studies
ICD	International Classification of Diseases
IQR	Interquartile Range
NHS	National Health Service
NSAIDs	Non-Steroidal Anti-Inflammatory Drugs
PAF	Population Attributable Fraction
PRS	Polygenic Risk Score
RCT	Randomized Controlled Trial
SD	Standard Deviation
SNP	Single Nucleotide Polymorphisms
