# Supplementary material for: Association of Non-Steroidal Anti-Inflammatory Drugs, Genetic Risk, and Environmental Risk Factors with Incidence of Colorectal Cancer
Source: Cancers (Basel). 2022 Oct 20;14(20):5138. doi: 10.3390/cancers14205138 (PMC9600467; doi:10.3390/cancers14205138)
Supplement: Supplementary file 1 [file cancers-14-05138-s001.zip › cancers-1891263 STROBE Checklist.pdf]

cancers MDPI

Supplementary Material: Association of Non-Steroidal Anti-Inflammatory Drugs, Genetic Risk, and Environmental Risk Factors with Incidence of Colorectal Cancer

Jiao-Jiao Ren, Pei-Dong Zhang, Zhi-Hao Li, Xi-Ru Zhang, Wen-Fang Zhong, Wei-Qi Song, Xing Wang, Ping-Ming Gao * and Chen Mao *

STROBE Statement—Checklist of items that should be included in reports of ***cohort studies***.

	Item No	Recommendation	Page No
**Title and abstract**	1	(*a*) Indicate the study's design with a commonly used term in the title or the abstract	Title and abstract
		(*b*) Provide in the abstract an informative and balanced summary of what was done and what was found	Abstract
		**Introduction**	
Background/rationale	2	Explain the scientific background and rationale for the investigation being reported	Introduction: paragraphs 1 and 3
Objectives	3	State specific objectives, including any prespecified hypotheses	Introduction: paragraphs 4
		**Methods**	
Study design	4	Present key elements of study design early in the paper	Introduction: paragraphs 3<br>Methods: Study Participants
Setting	5	Describe the setting, locations, and relevant dates, including periods of recruitment, exposure, follow-up, and data collection	Methods: Study Participants, Ascertainment of CRC Incidence
Participants	6	(*a*) Give the eligibility criteria, and the sources and methods of selection of participants. Describe methods of follow-up	Methods: Study Participants
		(*b*) For matched studies, give matching criteria and number of exposed and unexposed	N/a
Variables	7	Clearly define all outcomes, exposures, predictors, potential confounders, and effect modifiers. Give diagnostic criteria, if applicable	Methods: Polygenic risk score, Environmental risk score, Ascertainment of CRC Incidence<br>Supplement: Table S1 to 4
Data sources/ measurement	8*	For each variable of interest, give sources of data and details of methods of assessment (measurement). Describe comparability of assessment methods if there is more than one group	Methods: Polygenic risk score, Environmental risk score<br>Supplement: Table S1 to 4
Bias	9	Describe any efforts to address potential sources of bias	Methods: Statistical Analyses
Study size	10	Explain how the study size was arrived at	N/A
Quantitative variables	11	Explain how quantitative variables were handled in the analyses. If applicable, describe which groupings were chosen and why	Methods: Polygenic risk score, Environmental risk score
Statistical methods	12	(*a*) Describe all statistical methods, including those used to control for confounding	Methods: Statistical analysis
		(*b*) Describe any methods used to examine subgroups and interactions	Methods: Statistical analysis
		(*c*) Explain how missing data were addressed	Methods: Study Participants
		(*d*) If applicable, explain how loss to follow-up was addressed	Methods: Study Participants
		(*e*) Describe any sensitivity analyses	Methods: Statistical analysis
		**Results**	
Participants	13*	(a) Report numbers of individuals at each stage of study—eg numbers potentially eligible, examined for eligibility, confirmed eligible, included in the study, completing follow-up, and analysed	Methods: Study Participants<br>Result: paragraphs 1
		(b) Give reasons for non-participation at each stage	N/A
		(c) Consider use of a flow diagram	Figure S1
Descriptive data	14*	(a) Give characteristics of study participants (eg demographic, clinical, social) and information on exposures and potential confounders	Result: paragraphs 1<br>Table 1
		(b) Indicate number of participants with missing data for each variable of interest	Methods: Study Participants
		(c) Summarise follow-up time (eg, average and total amount)	Result: paragraphs 1
Outcome data	15*	Report numbers of outcome events or summary measures over time	Result: paragraphs 1

Main results	16	(*a*) Give unadjusted estimates and, if applicable, confounder-adjusted estimates and their precision (eg, 95% confidence interval). Make clear which confounders were adjusted for and why they were included	Result<br>Table 1 to 4
		(*b*) Report category boundaries when continuous variables were categorized	Methods: Polygenic risk score, Environmental risk score
		(*c*) If relevant, consider translating estimates of relative risk into absolute risk for a meaningful time period	Table 1 to 4
Other analyses	17	Report other analyses done—eg analyses of subgroups and interactions, and sensitivity analyses	Result
		**Discussion**	
Key results	18	Summarise key results with reference to study objectives	Discussion: paragraphs 1
Limitations	19	Discuss limitations of the study, taking into account sources of potential bias or imprecision. Discuss both direction and magnitude of any potential bias	Discussion: paragraphs 5
Interpretation	20	Give a cautious overall interpretation of results considering objectives, limitations, multiplicity of analyses, results from similar studies, and other relevant evidence	Discussion: paragraphs 2 to 5
Generalisability	21	Discuss the generalisability (external validity) of the study results	Discussion: paragraphs 1
		**Other information**	
Funding	22	Give the source of funding and the role of the funders for the present study and, if applicable, for the original study on which the present article is based	Funding/support

*Give information separately for exposed and unexposed groups.

Note: An Explanation and Elaboration article discusses each checklist item and gives methodological background and published examples of transparent reporting. The STROBE checklist is best used in conjunction with this article (freely available on the Web sites of PLoS Medicine at http://www.plosmedicine.org/, Annals of Internal Medicine at http://www.annals.org/, and Epidemiology at http://www.epidem.com/). Information on the STROBE Initiative is available at http://www.strobe-statement.org.
