# Supplementary material for: Association of Non-Steroidal Anti-Inflammatory Drugs, Genetic Risk, and Environmental Risk Factors with Incidence of Colorectal Cancer
Source: Cancers (Basel). 2022 Oct 20;14(20):5138. doi: 10.3390/cancers14205138 (PMC9600467; doi:10.3390/cancers14205138)
Supplement: Supplementary file 1 [file cancers-14-05138-s001.zip › cancers-1891263-supplementary tables and figure.pdf]

cancers MDPI

Supplementary Material: Association of Non-Steroidal Anti-Inflammatory Drugs, Genetic Risk, and Environmental Risk Factors with Incidence of Colorectal Cancer

Jiao-Jiao Ren, Pei-Dong Zhang, Zhi-Hao Li, Xi-Ru Zhang, Wen-Fang Zhong, Wei-Qi Song, Xing Wang, Ping-Ming Gao * and Chen Mao *

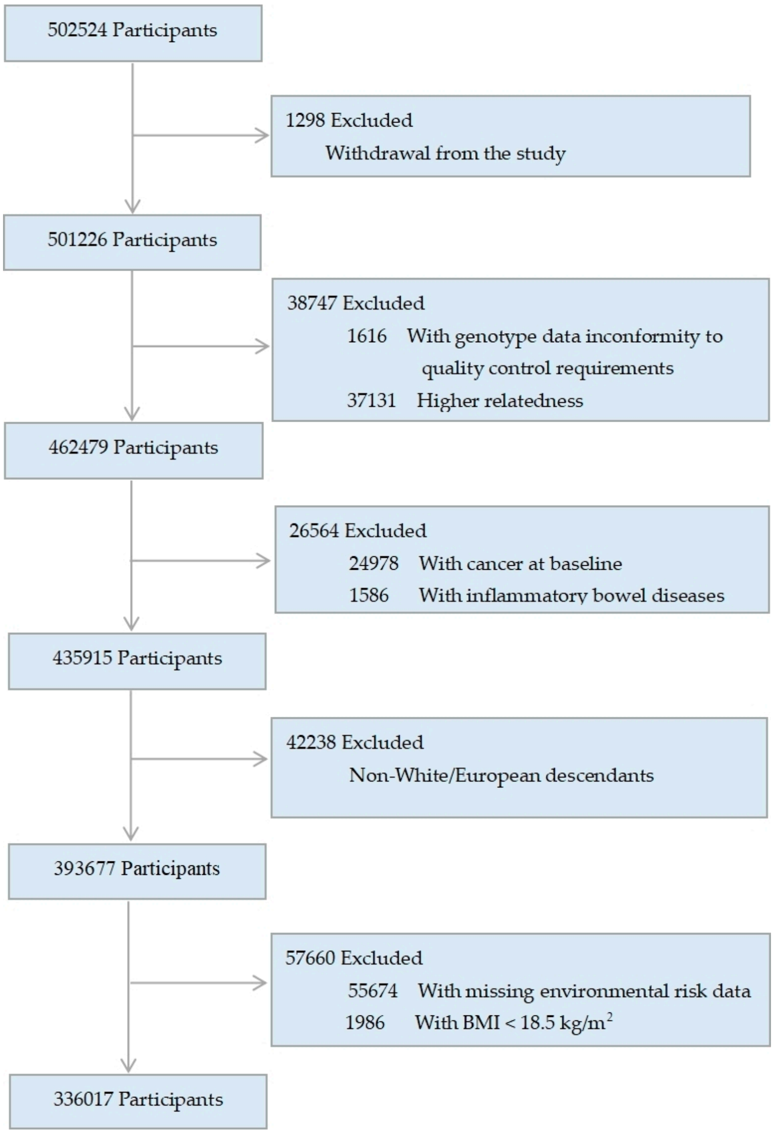

Figure S1. Flow diagram of the study population selection.

Table S1. List of single-nucleotide polymorphisms constructing the polygenic risk score for CRC.

SNP	Chromosome	Position	Non-risk allele	Risk allele	Risk allele frequency	β
rs4360494	1	38455891	C	G	0.4539	0.0379
rs12144319	1	55246035	T	C	0.2548	0.0661
rs72647484	1	22587728	C	T	0.9107	0.0504
rs7542665	1	62673037	T	C	0.273	0.0334
rs6678517	1	183002639	G	A	0.5898	0.073
rs17011141	1	222112634	A	G	0.2087	0.0877

SNP	Chromosome	Position	Non-risk allele	Risk allele	Risk allele frequency	β
rs448513	2	159964552	T	C	0.326	0.0054
rs11884596	2	199612407	T	C	0.3823	0.0342
rs983402	2	199781586	C	T	0.3312	0.0622
rs7606562	2	48686695	A	T	0.813	0.0414
rs11692435	2	98275354	A	G	0.9	0.0492
rs3731861	2	219191256	C	T	0.6295	0.0613
rs10049390	3	133701119	G	A	0.7353	0.0455
rs13086367	3	112903888	G	A	0.5262	0.0463
rs72942485	3	112999560	A	G	0.9802	0.0545
rs9831861	3	53088285	T	G	0.59	0.0294
rs35470271	3	40915239	A	G	0.154	0.0994
rs12635946	3	112916918	T	C	0.62	0.0334
rs113569514	3	133748789	C	T	0.62	0.0414
rs9876206	3	169517436	T	C	0.7507	0.0453
rs6781752	3	66365163	G	A	0.205	0.0597
rs11727676	4	145659064	T	C	0.098	0.0093
rs1391441	4	106128760	G	A	0.672	0.0148
rs13149359	4	94938618	C	A	0.3663	0.052
rs7708610	5	40102443	G	A	0.3564	0.0384
rs78368589	5	1240204	C	T	0.0597	0.0786
rs145364999	5	98206082	A	T	0.9969	0.3496
rs2735940	5	1296486	A	G	0.4952	0.0865
rs12514517	5	40280076	G	A	0.288	0.1013
rs755229494	5	112097351	A	G	0.0011	0.6286
rs12659017	5	125988175	A	G	0.232	0.0374
rs4976270	5	134467220	T	C	0.5501	0.0693
rs13204733	6	55566108	A	G	0.141	0.0643
rs116685461	6	31315512	A	G	0.8755	0.0655
rs9271695	6	32593080	A	G	0.7954	0.0889
rs2516420	6	31449620	T	C	0.9263	0.1091
rs116353863	6	31010185	T	C	0.0165	0.1202
rs16878812	6	35569562	G	A	0.8861	0.0778
rs9470361	6	36623379	G	A	0.2488	0.054
rs62404966	6	55712124	T	C	0.7623	0.0724
rs3131043	6	30758466	A	G	0.43	0.0294
rs2070699	6	12292772	G	T	0.48	0.0294
rs1476570	6	29809860	G	A	0.376	0.0492
rs3830041	6	32191339	C	T	0.14	0.0645
rs6928864	6	105966894	A	C	0.91	0.0531
rs62396735	6	41702582	T	C	0.2908	0.033
rs12672022	7	45136423	C	T	0.8345	0.0067
rs80077929	7	46094089	C	T	0.1107	0.0093
rs10951878	7	46926695	T	C	0.91	0.0531
rs3801081	7	47511161	A	G	0.49	0.0253
rs7013278	8	128414892	C	T	0.3761	0.0091
rs4313119	8	128571855	T	G	0.7486	0.0518
rs16892766	8	117630683	A	C	0.0829	0.2099
rs6469654	8	117632965	C	G	0.2288	0.0677
rs117079142	8	117790914	C	A	0.0432	0.1139
rs6983267	8	128413305	T	G	0.5228	0.1052
rs34405347	9	101679752	G	T	0.9034	0.0089
rs1537372	9	22103183	T	G	0.5692	0.012
rs10980628	9	113671403	T	C	0.2106	0.0511
rs12217641	10	8663875	T	C	0.6981	0.0069
rs10786560	10	101315166	A	G	0.762	0.0082
rs1250567	10	81046265	T	C	0.4405	0.047
rs11255841	10	8739580	A	T	0.703	0.1064
rs10821907	10	52648454	T	C	0.8276	0.073
rs704017	10	80819132	A	G	0.5846	0.0765
rs11190164	10	101351704	A	G	0.2626	0.0889
rs12246635	10	114288619	T	C	0.0983	0.0975
rs11196170	10	114722621	G	A	0.2178	0.0527
rs7946853	11	74409077	T	C	0.8624	0.0119
rs55864876	11	100717136	A	G	0.9184	0.015
rs2186607	11	101656397	A	T	0.5178	0.0483
rs61389091	11	74427921	T	C	0.9606	0.1934
rs4450168	11	10286755	A	C	0.17	0.0413

rs174533	11	61549025	A	G	0.6739	0.0636
rs7121958	11	74280012	T	G	0.5105	0.078
rs3087967	11	111156836	C	T	0.2911	0.1122
rs4759277	12	57533690	C	A	0.3546	0.0285
rs1427760	12	115100714	T	C	0.5268	0.0424
rs3217874	12	4400808	C	T	0.4282	0.0453
rs10849433	12	6406904	T	C	0.267	0.0468
rs11610543	12	43134191	A	G	0.5013	0.0474
SNP	Chromosome	Position	Non-risk allele	Risk allele	Risk allele frequency	β
rs35808169	12	4368607	T	C	0.1721	0.089
rs3217810	12	4388271	C	T	0.1253	0.1181
rs2250430	12	6421174	A	T	0.7095	0.0597
rs77969132	12	31594813	C	T	0.015	0.1583
rs12372718	12	51171090	A	G	0.3924	0.0896
rs597808	12	111973358	A	G	0.5166	0.0737
rs7300312	12	115890922	T	C	0.5719	0.066
rs2710310	12	12035649	T	C	0.7596	0.0145
rs78341008	13	73791554	T	C	0.0719	0.0109
rs8000189	13	111075881	C	T	0.6401	0.0473
rs45597035	13	73649152	G	A	0.6506	0.0495
rs1924816	13	73997961	G	A	0.7737	0.0506
rs7333607	13	37462010	A	G	0.235	0.0758
rs1330889	13	78609615	T	C	0.87	0.0453
rs1951864	14	54369299	G	A	0.3722	0.0059
rs17094983	14	59189361	A	G	0.8773	0.0062
rs8020436	14	59208437	G	A	0.4016	0.0294
rs35107139	14	54419106	A	C	0.4235	0.0912
rs4901473	14	54445157	A	G	0.378	0.0465
rs745213	15	68060389	T	G	0.8102	0.0072
rs12594720	15	67007018	G	C	0.7218	0.0246
rs56324967	15	67402824	T	C	0.6757	0.0689
rs17816465	15	33156386	G	A	0.2055	0.069
rs12708491	15	32992836	A	G	0.5872	0.0464
rs2293581	15	33010736	G	A	0.2116	0.1248
rs7495132	15	91172901	C	T	0.12	0.0453
rs9930005	16	80043258	A	C	0.4303	0.0061
rs12447408	16	86252544	G	A	0.2535	0.0079
rs9924886	16	68743939	C	A	0.7321	0.055
rs12149163	16	86339315	C	T	0.4976	0.0487
rs62042090	16	86703949	C	T	0.2164	0.0481
rs983318	17	70413253	G	A	0.2526	0.0397
rs73975586	17	814243	T	A	0.8732	0.0497
rs1078643	17	10707241	G	A	0.7636	0.0747
rs75954926	17	81061048	A	G	0.6568	0.0882
rs373585858	17	80394556	G	A	0.0016	0.1103
rs4968127	17	809643	A	G	0.3684	0.0514
rs11874392	18	46453156	T	A	0.545	0.1606
rs73068325	19	59079096	C	T	0.1826	0.0066
rs34797592	19	16417198	C	T	0.1182	0.0824
rs28840750	19	33519927	G	T	0.948	0.1939
rs1963413	19	41871573	G	A	0.6119	0.0441
SNP	Chromosome	Position	Non-risk allele	Risk allele	Risk allele frequency	β
rs12979278	19	49218602	C	T	0.53	0.0293
rs2738783	20	62308612	G	T	0.2029	0.006
rs6067417	20	48983697	T	C	0.5635	0.0331
rs6031311	20	42666475	C	T	0.7591	0.0362
rs6091189	20	49256285	C	T	0.1529	0.0549
rs994308	20	6603622	T	C	0.5939	0.0626
rs28488	20	6762221	C	T	0.6388	0.0714
rs556532366	20	8568071	C	T	0.0029	0.0715
rs189583	20	6376457	C	G	0.3298	0.0795
rs4813802	20	6699595	T	G	0.3561	0.0819
rs11087784	20	7740976	A	G	0.1523	0.0874
rs6066825	20	47340117	G	A	0.6448	0.0719
rs6063514	20	49055318	T	C	0.6086	0.0547
rs13831	20	57475191	A	G	0.684	0.0334
rs1741640	20	60932414	T	C	0.7652	0.1146

rs6058093	20	33213196	A	C	0.4942	0.045

Table S2. Definitions of environmental risk factors in the UK Biobank[a] .

Factors	Definitions	Field ID of UK biobank
Education	Education was categorized into two groups: the high qualifications included 'College or university degree', 'NVQ or HND or HNC or equivalent', 'Other professional qualifications', and 'A levels/AS levels or equivalent'; the others defined as low qualifications.[1]	6138
DASH score (diet)	According to the DASH recommendation: with regard to intake of fruits, vegetables, whole grains, and low-fat dairy products, participants with the 1 quintile (lowest) get 1 score and those with the 5 quintile (highest) get 5 score; on the contrary, consumption of red or processed meats, sugar-sweetened beverages, and sodium, participants with the 1 quintile (lowest) get 5 score and the 5 quintile (highest) get 1 score.[2,3]	1309, 1319, 1289, 1299, 1438, 1448, 1458, 1468, 1408, 1418, 1349, 3680, 1359, 1369, 1379, 1389, 3680, 6144, 1478
Smoking	Smoking combined smoking status with smoking pack-years, and defined as two groups: current or former (≥ 30 pack years) and never or former (< 30 pack years).[4,5]	20116, 2867, 2887, 2897, 2907, 3436, 3486, 6183, 6194, 20161
Alcohol consumption	Based on Dietary Guidelines for Americans: 1 drink equivalent is equivalent to 14g of pure alcohol.[6]	20117, 1558, 1568, 1578, 1588, 1598, 1608, 5364, 4407, 4418, 4429, 4440, 4451, 4462
Physical activity	Based on Global Recommendations on Physical Activity for Health: Regular physical activity was described as greater than or equal to moderate activity for 150 minutes, or vigorous activity for 75 minutes every week, or the combination of moderate and vigorous for 150 minutes.[7]	884, 894, 904, 914
Occupational exposure	Frequency of exposure to materials that included asbestos, paints, thinners, glues, pesticides, or other fumes at work.	22610, 22612, 22613, 22614
BMI	Weight in kilograms divided by height in meters squared	21001

[a] BMI, body mass index; DASH, Dietary Approaches to Stop Hypertension.

Table S3. Descriptions of environmental risk factors used to derive unweighted environmental risk score[a.]

Environmental risk factors	Points	Descriptions
Education	0	Low qualification
	1	High qualification
DASH score (diet)	0	Unhealthy diet quality: DASH score < 24[b]
	1	Healthy diet quality: DASH score ≥ 24
Smoking	0	Current or former (≥ 30 pack years)
	1	Never or former (< 30 pack years)
Alcohol consumption	0	> 24 g/day for men, > 12 g/day for women[c]
	1	≤ 24 g/day for men, ≤ 12g/day for women
Physical activity	0	Irregular physical activity
	1	Regular physical activity
Occupational exposure	0	Expose to materials containing asbestos, paints, thinners, glues, pesticides, or other fumes at work
	1	Expose none
History of type 2 diabetes	0	Yes
	1	No
BMI	0	≥ 25 kg/m^2 (overweight or obese)[d]
	1	18.5 < BMI < 25 kg/m^2 (healthy weight)

[a] CRC, colorectal cancer; BMI, body mass index; DASH, Dietary Approaches to Stop Hypertension.

[b] DASH score in the highest 40%;.

[c] Recommendation on alcohol consumption based on World Cancer Research Fund/American Institute for Cancer Research (WCRF/AICR);.

[d] Recommendation on obesity based on the World Health Organization.

Table S4. Definitions of diseases in the UK Biobank[a.]

Diseases	ICD-9	ICD-10	Self-reported fields
CRC	153, 154.0, 154.1	C18, C19, C20	20001 (1020, 1022, 1023)
Type 2 diabetes	250	E11	2443 (1), 2976, 6153 (3), 6177 (3), 20002 (1223)
Crohn's disease	555	K50	131627, 20002 (1462)
Ulcerative colitis	556	K51	131629, 20002 (1463)

[a] ICD, International Classification of Diseases; CRC, colorectal cancer.

Table S5. The hazard ratios of CRC incidence risk associated with each environmental risk factor and the environmental risk score[a.]

	HR (95% CI)[b]	P value
Environmental risk factors		
Low education level	1.14 (1.06-1.23)	0.001
Overweight or obese (BMI ≥25 kg/m^2)	1.16 (1.07-1.26)	< 0.001
Current or former (≥ 30 pack years) smokers	1.40 (1.27-1.54)	0.002
Excessive alcohol consumption	1.31 (1.19-1.44)	< 0.001
Unhealthy diet (DASH score)	1.13 (1.05-1.22)	0.001
Irregular physical activity	1.08 (1.00-1.16)	0.048
Occupational exposure	1.16 (1.06-1.27)	0.001
History of type 2 diabetes	1.45 (1.27-1.67)	< 0.001
Environmental risk score	1.29 (1.23-1.36)	< 0.001

[a] CRC, colorectal cancer; BMI, body mass index; DASH, Dietary Approaches to Stop Hypertension; CI, confidence interval.

[b] Adjusted for age, sex, household income, Townsend deprivation index, family history of CRC, screening history of CRC, non-steroidal anti-inflammatory drugs, relatedness, genotyping chip, first 20 principal components of ancestry, and the remaining environmental risk factors.

Table S6. The CRC incidence risk in accordance with environmental risk category within each genetic risk category[a.]

Genetic risk	Environmental risk	No. of participants	No. of CRC cases (%)/Person-years	HR (95% CI)[b]	P value	P value for trend	P value for interaction[c]
Low genetic risk	Low environmental risk	25507	102 (0.40)/227013	1 [reference]			
Low genetic risk	Intermediate environmental risk	28490	118 (0.41)/253392	0.94 (0.72-1.22)	0.634	0.710	
Low genetic risk	High environmental risk	13209	73 (0.55)/113188	1.08 (0.79-1.46)	0.639		
Intermediate genetic risk	Low environmental risk	76336	493 (0.65)/679657	1 [reference]			
Intermediate genetic risk	Intermediate environmental risk	85018	746 (0.88)/757585	1.22 (1.09-1.37)	0.001	< 0.001	0.131
Intermediate genetic risk	High environmental risk	40254	429 (1.07)/344747	1.34 (1.18-1.53)	< 0.001		
High genetic risk	Low environmental risk	25392	278 (1.09)/219251	1 [reference]			
High genetic risk	Intermediate environmental risk	28515	437 (1.53)/242750	1.28 (1.10-1.48)	0.002	< 0.001	
High genetic risk	High environmental risk	13296	266 (2.00)/110122	1.54 (1.30-1.83)	< 0.001		

[a] CRC, colorectal cancer; HR, hazard ratio; CI, confidence interval.

[b] Cox proportional hazards regression adjusted for age, sex, household income, Townsend deprivation index, family history of CRC, screening history of CRC, non-steroidal anti-inflammatory drug use, relatedness, genotyping chip, and first 20 principal components of ancestry; P value for trend calculated considering each environmental risk category as continuous variables.

[c] The interaction between the polygenic risk score and the weighted environmental risk score.

Table S7. The CRC incidence risk in accordance with the combined genetic risk and environmental risk[a.]

Genetic risk	Environmental risk	No. of participants	No. of CRC cases (%)/Person-years	HR (95% CI)[b]	P value	P value for trend
Low genetic risk	Low environmental risk	25507	102 (0.40)/227013	1 [reference]		
Low genetic risk	Intermediate environmental risk	28490	118 (0.41)/253392	0.95 (0.73-1.23)	0.687	
Low genetic risk	High environmental risk	13209	73 (0.55)/113188	1.13 (0.84-1.53)	0.430	
Intermediate genetic risk	Low environmental risk	76336	493 (0.65)/679657	1.60 (1.29-1.98)	< 0.001	
Intermediate genetic risk	Intermediate environmental risk	85018	746 (0.88)/757585	1.94 (1.58-2.39)	< 0.001	< 0.001
Intermediate genetic risk	High environmental risk	40254	429 (1.07)/344747	2.22 (1.79-2.76)	< 0.001	
High genetic risk	Low environmental risk	25392	278 (1.09)/219251	2.73 (2.17-3.42)	< 0.001	
High genetic risk	Intermediate environmental risk	28515	437 (1.53)/242750	3.35 (2.70-4.16)	< 0.001	
High genetic risk	High environmental risk	13296	266 (2.00)/110122	4.18 (3.32-5.26)	< 0.001	

[a] CRC, colorectal cancer; HR, hazard ratio; CI, confidence interval.

[b] Cox proportional hazards regression adjusted for age, sex, household income, Townsend deprivation index, family history of CRC, screening history of CRC, non-steroidal anti-inflammatory drug use, relatedness, genotyping chip, and first 20 principal components of ancestry; P value for trend calculated considering each environmental risk category as continuous variables.

Table S8. The CRC incidence risk in accordance with the combined genetic and environmental risk according to NSAIDs use after excluding related participants, events occurred within the first 2 years and individuals with missing covariate data[a].

Subgroup	Related participants excluded[b] (n=293517)		Outcomes within 2 years excluded[c] (n=335591)		Missing covariate data excluded[c] (n=293309)	
	HR (95% CI)	P value	HR (95% CI)	P value	HR (95% CI)	P value
Non-regular use of NSAIDs						
Low genetic risk						
Low environmental risk	1 [reference]		1 [reference]		1 [reference]	
Intermediate environmental risk	0.90 (0.66-1.24)	0.530	0.90 (0.66-1.24)	0.532	1.01 (0.61-1.67)	0.979
High environmental risk	1.17 (0.83-1.67)	0.368	1.18 (0.83-1.67)	0.366	0.86 (0.45-1.67)	0.666
Intermediate genetic risk						
Low environmental risk	1.63 (1.27-2.10)	< 0.001	1.63 (1.27-2.10)	< 0.001	1.67 (1.15-2.47)	< 0.001
Intermediate environmental risk	2.15 (1.72-2.69)	< 0.001	2.14 (1.72-2.69)	< 0.001	2.21 (1.66-3.05)	< 0.001
High environmental risk	2.56 (2.05-3.12)	< 0.001	2.53 (2.02-3.10)	< 0.001	2.60 (1.81-3.84)	< 0.001
High genetic risk						
Low environmental risk	2.85 (2.18-3.72)	< 0.001	2.82 (2.16-3.69)	< 0.001	2.89 (1.91-4.40)	< 0.001
Intermediate environmental risk	3.41 (2.64-4.41)	< 0.001	3.39 (2.63-4.39)	< 0.001	3.42 (2.24-5.21)	< 0.001
High environmental risk	4.48 (3.42-5.85)	< 0.001	4.46 (3.41-5.84)	< 0.001	4.51 (3.24-6.61)	< 0.001
Regular use of NSAIDs						
Low genetic risk						
Low environmental risk	1 [reference]		1 [reference]		1 [reference]	
Intermediate environmental risk	1.08 (0.66-1.78)	0.748	1.09 (0.66-1.78)	0.741	1.16 (0.69-1.94)	0.577
High environmental risk	1.02 (0.54-1.92)	0.947	1.02 (0.55-1.92)	0.941	1.06 (0.54-2.07)	0.868
Intermediate genetic risk						
Low environmental risk	1.58 (1.05-2.38)	0.027	1.57 (1.05-2.36)	0.029	1.60 (1.04-2.48)	0.013
Intermediate environmental risk	2.05 (1.38-3.04)	< 0.001	2.03 (1.37-3.02)	< 0.001	2.09 (1.33-3.27)	< 0.001
High environmental risk	2.35 (1.54-3.58)	< 0.001	2.31 (1.52-3.52)	< 0.001	2.40 (1.47-3.90)	< 0.001
High genetic risk						
Low environmental risk	2.50 (1.61-3.88)	< 0.001	2.48 (1.59-3.86)	< 0.001	2.55 (1.55-4.12)	< 0.001
Intermediate environmental risk	3.35 (2.22-5.08)	< 0.001	3.33 (2.20-5.04)	< 0.001	3.41 (2.13-5.40)	< 0.001
High environmental risk	3.54 (2.23-5.62)	< 0.001	3.55 (2.23-5.63)	< 0.001	3.57 (2.18-6.14)	< 0.001

[a] CRC, colorectal cancer; HR, hazard ratio; CI, confidence interval; NSAIDs, non-steroidal anti-inflammatory drugs.

[b] Adjusted for age, sex, household income, Townsend deprivation index, family history of CRC, screening history of CRC, genotyping chip, and first 20 principal components of ancestry.

[c] Adjusted for age, sex, household income, Townsend deprivation index, family history of CRC, screening history of CRC, relatedness, genotyping chip, and first 20 principal components of ancestry

Table S9. The CRC incidence risk in accordance with the combined genetic and environmental risk according to NSAIDs use by sex and age[a].

Subgroup	Male[b]		Female[b]		Age < 60 years[c]		Age ≥ 60 years[c]	
	HR (95% CI)	P value	HR (95% CI)	P value	HR (95% CI)	P value	HR (95% CI)	P value
Non-regular use of NSAIDs								
Low genetic risk								
Low environmental risk	1 [reference]		1 [reference]		1 [reference]		1 [reference]	
Intermediate environmental risk	1.40 (0.88-2.22)	0.152	0.56 (0.35-1.21)	0.518	0.76 (0.45-1.28)	0.298	1.02 (0.69-1.51)	0.931
High environmental risk	1.67 (1.02-2.72)	0.040	0.85 (0.49-1.47)	0.562	0.64 (0.31-1.32)	0.228	1.50 (0.99-2.26)	0.054
Intermediate genetic risk								
Low environmental risk	1.99 (1.29-2.92)	0.002	1.40 (1.02-1.93)	0.037	1.87 (1.28-2.56)	0.010	1.61 (1.16-2.22)	0.004
Intermediate environmental risk	2.61 (1.76-3.87)	< 0.001	1.42 (1.01-1.96)	0.029	2.49 (1.92-3.34)	0.001	2.00 (1.46-2.73)	< 0.001
High environmental risk	3.29 (2.21-4.90)	< 0.001	1.96 (1.57-2.51)	0.008	2.79 (2.09-3.84)	< 0.001	2.33 (1.69-3.23)	< 0.001
High genetic risk								
Low environmental risk	3.51 (2.29-5.38)	< 0.001	2.44 (1.77-3.39)	< 0.001	2.96 (1.98-4.30)	< 0.001	2.80 (2.01-3.99)	< 0.001

Intermediate environmental risk	4.61 (3.08-6.90)	< 0.001	3.15 (2.12-4.27)	< 0.001	3.53 (2.37-5.26)	< 0.001	3.26 (2.35-4.53)	< 0.001
High environmental risk	6.01 (3.98-9.08)	< 0.001	3.36 (2.32-4.86)	< 0.001	4.94 (3.25-7.52)	< 0.001	4.17 (2.96-5.88)	< 0.001
P value for interaction	0.428				0.112			
Regular use of NSAIDs								
Low genetic risk								
Low environmental risk	1 [reference]		1 [reference]		1 [reference]		1 [reference]	
Intermediate environmental risk	1.31 (0.66-2.62)	0.443	0.90 (0.43-1.91)	0.793	1.01 (0.38-2.69)	0.989	1.10 (0.62-1.96)	0.738
High environmental risk	1.15 (0.50-2.66)	0.747	0.98 (0.36-2.69)	0.971	1.32 (0.40-4.41)	0.648	0.94 (0.45-1.96)	0.866
Intermediate genetic risk								
Low environmental risk	1.97 (1.07-3.62)	0.030	1.29 (0.74-2.23)	0.365	1.78 (0.84-3.78)	0.135	1.51 (0.93-2.45)	0.094
Intermediate environmental risk	2.35 (1.30-4.25)	0.005	1.58 (0.84-2.99)	0.159	2.38 (1.14-5.00)	0.022	1.93 (1.21-3.08)	0.006
High environmental risk	3.02 (1.64-5.55)	< 0.001	1.88 (1.09-3.22)	0.022	2.66 (1.18-5.97)	0.018	2.23 (1.36-3.65)	0.001
High genetic risk								
Low environmental risk	2.80 (1.44-5.43)	0.002	2.39 (1.33-4.28)	0.003	2.76 (1.23-6.21)	0.014	2.53 (1.50-4.26)	< 0.001
Intermediate environmental risk	3.90 (2.12-7.20)	< 0.001	3.12 (1.76-5.54)	< 0.001	3.44 (1.74-7.33)	< 0.001	2.80 (1.71-4.61)	< 0.001
High environmental risk	5.23 (2.76-9.91)	< 0.001	3.29 (1.85-6.45)	< 0.001	4.81 (2.01-11.52)	< 0.001	3.35 (1.95-5.75)	< 0.001
P value for interaction	0.625				0.025			

[a] CRC, colorectal cancer; HR, hazard ratio; CI, confidence interval; NSAIDs, non-steroidal anti-inflammatory drugs.

[b] Adjusted for age, household income, Townsend deprivation index, family history of CRC, screening history of CRC, relatedness, genotyping chip, and first 20 principal components of ancestry.

[c] Adjusted for sex, household income, Townsend deprivation index, family history of CRC, screening history of CRC, relatedness, genotyping chip, and first 20 principal components of ancestry.

Table S10. The CRC incidence risk in accordance with the combined genetic and environmental risk according to NSAIDs use by screening history of CRC[a].

Subgroup	Screening history[b]		No screening history[b]	
	HR (95% CI)	P value	HR (95% CI)	P value
Non-regular use of NSAIDs				
Low genetic risk				
Low environmental risk	1 [reference]		1 [reference]	
Intermediate environmental risk	1.22 (0.69-2.16)	0.495	0.77 (0.53-1.13)	0.185
High environmental risk	1.34 (0.71-2.54)	0.363	1.10 (0.73-1.66)	0.640
Intermediate genetic risk				
Low environmental risk	1.60 (0.98-2.63)	0.061	1.60 (1.19-2.13)	0.002
Intermediate environmental risk	2.00 (1.21-3.31)	0.003	2.15 (1.60-2.66)	< 0.001
High environmental risk	2.04 (1.27-3.29)	0.007	2.75 (2.18-3.52)	< 0.001
High genetic risk				
Low environmental risk	2.22 (1.93-5.38)	< 0.001	2.91 (2.21-3.87)	< 0.001
Intermediate environmental risk	3.19 (1.87-5.36)	< 0.001	3.49 (2.65-4.62)	< 0.001
High environmental risk	4.05 (2.40-6.86)	< 0.001	4.44 (3.27-6.05)	< 0.001
P value for interaction		0.499		
Regular use of NSAIDs				
Low genetic risk				
Low environmental risk	1 [reference]		1 [reference]	
Intermediate environmental risk	0.96 (0.41-2.23)	0.925	1.14 (0.62-2.10)	0.674
High environmental risk	0.73 (0.23-2.35)	0.602	1.17 (0.55-2.49)	0.681
Intermediate genetic risk				
Low environmental risk	1.36 (0.68-2.71)	0.389	1.51 (1.02-2.64)	0.046
Intermediate environmental risk	1.48 (0.76-2.91)	0.253	2.05 (1.24-3.54)	0.001
High environmental risk	1.38 (0.64-2.94)	0.409	2.15 (1.11-4.06)	< 0.001
High genetic risk				
Low environmental risk	2.21 (1.05-4.68)	0.038	2.58 (1.42-4.56)	< 0.001
Intermediate environmental risk	2.41 (1.04-5.62)	0.041	3.44 (2.06-6.27)	< 0.001
High environmental risk	2.46 (1.21-5.03)	0.013	4.29 (2.46-7.47)	< 0.001

P value for interaction	0.376

[a] CRC, colorectal cancer; HR, hazard ratio; CI, confidence interval; NSAIDs, non-steroidal anti-inflammatory drugs.

[b] Adjusted for age, household income, Townsend deprivation index, family history of CRC, relatedness, genotyping chip, and first 20 principal components of ancestry.

References

1. Chadeau-Hyam M, Bodinier B, Vermeulen R, Karimi M, Zuber V, Castagné R, Elliott J, Muller D, Petrovic D, Whitaker M, et al. Education, biological ageing, all-cause and cause-specific mortality and morbidity: UK biobank cohort study. EClinicalMedicine 2020;29-30:100658. doi: 10.1016/j.eclinm.2020.100658.
2. Fung TT, Chiuve SE, McCulloguh ML, Rexrode KM, Logroscino G, Hu FB. Adherence to a DASH-style diet and risk of coronary heart disease and stroke in women. Arch Intern Med 2008;168:713-20. doi: 10.1001/archinte.168.7.713.
3. Fung TT, Hu FB, Wu K, Chiuve SE, Fuchs CS, Giovannucci E. The Mediterranean and Dietary Approaches to Stop Hypertension (DASH) diets and colorectal cancer. Am J Clin Nutr 2010;92:1429-35. doi: 10.3945/ajcn.2010.29242.
4. Tsoi KK, Pau CY, Wu WK, Chan FK, Griffiths S, Sung JJ. Cigarette smoking and the risk of colorectal cancer: a meta-analysis of prospective cohort studies. Clin Gastroenterol Hepatol 2009;7(6):682-8.e1-5. doi: 10.1016/j.cgh.2009.02.016.
5. Walter V, Jansen L, Hoffmeister M, Ulrich A, Chang-Claude J, Brenner H. Smoking and survival of colorectal cancer patients: population-based study from Germany. Int J Cancer 2015;137(6):1433-45. doi: 10.1002/ijc.29511.
6. Dietary Guidelines for Americans, 2015-2020: National Nutrition Guideline, 2019.
7. World Health Organization. Global Recommendations on Physical Activity for Health. Geneva, 2010.
